# Crisis-repair sequences - considerations on the classification and assessment of breaches in the therapeutic relationship

**DOI:** 10.1186/1471-2288-12-10

**Published:** 2012-02-03

**Authors:** Antje Gumz, Elmar Brähler, Michael Geyer, Rainer Erices

**Affiliations:** 1 Department of Psychosomatic Medicine and Psychotherapy, University of Leipzig, Semmelweisstraβe 10, Leipzig, 04103, Germany; 2 Department of Medical Psychology and Medical Sociology, University of Leipzig, Philipp-Rosenthal-Straβe 55, Leipzig, 04103, Germany; 3 Academy for Psychotherapy Erfurt, Fischmarkt 5, Erfurt, 99084, Germany; 4 Institute for History of Medicine and Medical Ethics of the University Erlangen-Nürnberg, Glückstraβe 10, Erlangen, 91054, Germany

## Abstract

**Background:**

Recent research indicates that temporary deteriorations of variables monitored continuously in the course of the therapeutic relationship are important characteristics of psychotherapeutic change. These so-called rupture-repair episodes were assessed by different authors using different mathematical methods.

**Methods:**

The study deals with the criteria for identifying rupture-repair episodes that have been established in previous studies. It proposes modifications of these criteria which prospectively could make it possible to identify rupture-repair episodes more precisely and consistently. The authors developed an alternative criterion. This criterion is able to include crisis patterns which had not been considered before, as well as to characterize the length of the crises. As a sample application, the different criteria were applied to continuously measured assessments of the therapeutic interaction in psychodynamic therapy courses (ten shorter processes and one long-term therapy).

**Results:**

The analysis revealed that the number of the identified rupture-repair episodes differed depending on the criterion that was used. Considerably more crises were identified with the newly developed criterion. The authors developed a classification of crisis patterns. They distinguished five patterns of crises and their resolution in therapy processes and ascertained the frequency of distribution. The most frequent pattern was the simple V-shape. The second most common pattern was a decline over more than one session with a sudden repair. The longest downward trend comprised a period of six sessions.

**Conclusions:**

The findings of the study give insight into basic mechanisms of change within the therapeutic relationship. A phenomenological discussion of how a crisis is defined is useful to create a methodological approach to the operationalization of crises, to differentiate specific characteristics and to specifically link these characteristics to the outcome in future studies. The methodological deliberations might be applyable to different research areas where the analysis of fluctuations in a variable of interest over time is relevant.

## Background

Numerous studies have provided evidence linking the quality of the therapeutic relationship to the outcomes of all treatment modalities [1,2]. The studies relate to different components of the therapeutic relationship such as the working alliance, the transference-countertransference configuration, or the real relationship [3,4].

It could be derived that any deterioration of the therapeutic relationship in the process is a bad thing, but this is not necessarily so. Studies have proven the positive importance of temporary crises in the therapeutic relationship for the outcome. Various different terms are used to refer to these crises. Many researchers used the term "rupture-repair episodes" [5]. In the context of qualitative retrospective studies they were, for example, referred to as "impasses" and "misunderstanding events" [6,7].

Safran et al. [5,8] discussed at length the importance of investigating alliance ruptures. They suggested that alliance ruptures inevitably occur in treatment courses and that these breaches can provide an opportunity for therapeutic change. Crises have been investigated over different time units. Some authors focused on crisis events occurring within a single session. They measured alliance ruptures either directly (assessments by patients and therapists) [9,10], indirectly, via instruments measuring aspects of the therapeutic relationship [5], or with the aid of external observers [5,11,12].

Far less authors examined the developments of the therapeutic relationship over a longer period of the therapeutic process. They focused on crisis events by measuring temporary sudden deteriorations in the trajectories of therapeutic relationship variables [13,14]. These variables were monitored over the course of psychotherapy processes using post-session patients self-assessments. These studies were influenced by researchers who had investigated the course of the therapeutic relationship on the basis of aggregated data by applying cluster analysis procedures: Different change profiles of alliance ratings and their connection to the therapeutic outcome are described in the literature [15,16]. Kivlighan and Shaughnessy [17] found three patterns of alliance development in the course of counseling processes: the stable, linear and quadratic patterns. The quadratic pattern correlated with a positive outcome.

Stiles et al. [13] wanted to replicate the latter findings on the alliance curves of 79 depressed patients (psychodynamic interpersonal and cognitive-behavioral short-term therapies, 2 groups - 8 or 16 sessions). They revealed that V-shaped strong deteriorations with subsequent improvements to the previous or a higher level (alliance rupture-repair episodes) proved crucial in their sample, rather than the quadratic pattern. They showed that patients whose course of alliance was characterized by rupture-repair sequences made larger gains in treatment compared to other patients. In 2006, Strauss et al. [14] observed alliance rupture-repair episodes in 30 patients with obsessive-compulsive and avoidant personality disorder in cognitive-behavioral courses of therapy. They ascertained that most of the patients who reported rupture-repair episodes also reported symptom reductions of 50% or more in all outcome measures.

Other authors have included the theories of nonlinear systems and self-organization to examine the developments of the therapeutic relationship over the entire process and to conceptualize crises. They characterized the therapeutic relationship as a complex, self-organizing system with stable and unstable episodes and abrupt transitions [18,19]. From this theoretical point of view, they defined a crisis as a period of varying length in which the system elements become increasingly unstable. The authors [19] registered a temporal connection between abrupt improvements to a higher mean score of interaction variables and local instability maxima and noticed that the instable episodes in each case were characterized by one or more pronounced negative slumps. Gumz, Bauer, and Brähler [20] assumed that the patient and therapist constitute a joint relationship system in which crises are experienced together simultaneously. Destabilization of post-session ratings was highly synchronous and the level of destabilization corresponded highly in successful therapies.

In the context with the above-mentioned topics studies focusing on symptom courses are worth mentioning. There is considerable evidence to suggest that symptomatic change within psychotherapeutic processes occurs in a nonlinear, discontinuous manner. Different types of discontinuous change are described in the literature. These include abrupt improvements (mostly referred to as "sudden gains" or „rapid early responses") and deteriorations ("sudden losses") from one session to another (for review see [21,19]). Another type of discontinuous change is V-shaped, representing an abrupt deterioration in the trajectory of measured variables followed by a subsequent return to the previous or a higher level. Hayes et al. [22] observed this type of discontinuity in addition to "sudden gains" in the progression of depressive symptoms trajectories. This type is equivalent to the rupture repair episodes observed in the course of variables of the therapeutic relationship as described by Stiles et al. [13] and Strauss et al. [14]. The majority of authors have demonstrated a connection between discontinuous changes in the trajectories of symptoms and positive therapeutic outcome.

### An open research question: How much crisis can the therapeutic relationship tolerate?

As mentioned above, both a good therapeutic relationship and temporary breaches are associated with a good treatment outcome. The resulting question is: How much crisis can and should the therapeutic relationship tolerate? Or to put it another way: What crises are useful which ones should be deemed harmful to the process?

The aforementioned studies have addressed specific phenomena of crises occuring within the process of therapy and linked these phenomena to the therapeutic outcome. A detailed model of the in-session rupture-resolution-process occurring in single sessions was elaborated [5]. It remains to be investigated, whether the model can be transferred to crises exceeding one session. At this stage of research, we believe it is necessary to find a systematic approach to investigate crises phenomena lasting over more than one session. A phenomenological discussion of the conception of crises is useful in order to clearly distinguish specific aspects and to specifically link these characteristics to the treatment outcome in future studies. The advantage of this kind of systematic research is that it will be possible prospectively, to draw conclusions about positive as well as negative correlations.

Crises can be measured either directly (direct questions about the presence of a rupture in the therapeutic relationship and their resolution [9,10]) or indirectly, via instruments measuring the quality of the therapeutic relationship (on the basis of differences in the values of these relationship variables [5,13,14]). The assessments of the presence of a rupture or of the quality of the therapeutic relationship can be made by patients and by therapists [9,10,13,14] or by external observers [5,11,12]. We can either focus on crisis events occurring within single sessions (ratings of the session process [5,9-12]) or on crises extending over a longer period of time (more than one or multiple sessions [13,14]). Investigations of the overall process are based on the aggregated information of what was going on in the single sessions.

In the context of the present article, we only refer to the identification of crises on the basis of the differences in the value of variables monitored over the course of psychotherapy processes using post-session self-assessments.

We assume that therapeutic crises in the process basically can be distinguished with regard to three characteristics: magnitude, number and length. Previous studies assessed, whether a crisis was present or not. These so-called rupture-repair episodes were assessed by different authors using different mathematical methods. The mathematical criteria differed with respect to the threshold for identifying crises. The number of crises that were identified as such depended on whether they reached a certain magnitude that had been pre-defined. Moreover, the aspect of the length of the crises has not been taken into account in previous studies. To analyse this aspect in detail, it is advisable to collect variables as frequently as possible throughout the process. In the study of Strauss et al. [14], the questionnaire was administered up to eight times (at least three times), at sessions 2, 5, 10, 20, 30, 40, 50, and 52. In the study of Stiles et al. [13], clients completed the questionnaire after each session.

### Aims of the present study

In the context of our process research, we closely explored every detail of individual process characteristics in the course of psychodynamic processes. One or more temporary and more or less pronounced deteriorations in the experienced therapeutic interaction played a role in the majority of the processes. The above-mentioned authors who examined the "rupture-repair" episodes in the course of variables of the therapeutic alliance [7,8] used different criteria to identify these crises. We found differences in the mathematical construction of these two criteria which made it difficult to decide which of them is better suited for selecting the most relevant rupture-repair episodes in the analyzed curves. We noticed that the criteria do not fully capture specific patterns of change. In addition, when attempting to apply the criteria, we found that certain criteria definitions showed potential deficiencies. From these observations and preliminary considerations we derived the objectives of the present study:

(a) We explain and compare the methods previously used for measuring rupture-repair episodes in longitudinal data [7,8]. We demonstrate the differences between the criteria using several courses of individual psychodynamic therapy as an example. We consider modifications of these criteria in order to standardize the procedures.

(b) We suggest an alternative criterion. Using this criterion it is possible to include crisis patterns which had not been identified before as well as to characterize the length of the crises.

(c) We classify different patterns of crises and their resolutions that may occur in therapy processes. We examine the frequency at which these patterns occur and the length of the episodes.

## Methods

### Procedure

First, we theoretically investigated the existing methods for measuring crises. We compared the definitions of Stiles et al. and Strauss et al. We applied their criteria on newly collected data, an example of ten shorter and one longer therapy courses, in order to demonstrate the effect of using different definitions for the identification of crises. We analyzed the criteria regarding potential deficiencies and considered possibilities for their modification. Secondly, we developed an alternative criterion to be able to include crisis patterns which had not been identified before. We applied this criterion on our data to get an idea about the effect of broadening the crisis concept on the number of identified crises. Finally, we developed a classification of crisis patterns and ascertained the frequency of distribution of these types.

All measurements and calculations on the basis of the described sample are intended to illustrate our considerations. As we do not aim to draw clinical conclusions from the calculations, we keep the description of the sample and measurement instrument short.

### Sample

The sample consists of ten courses of depth psychology-based psychotherapy with 29 to 35 sessions (subsample 1) and one 200-session extract from an analytic long-term therapy course (subsample 2). (In German-speaking regions the term "depth psychology-based psychotherapy" is used to refer to an application of the psychoanalytic method characterized by a limitation of the treatment goal, a primarily conflict-centered approach and a restriction of regressive processes. In the international nomenclature, the term corresponds to psychodynamic therapy.) Patients were diagnosed according to ICD-10-GM definitions [23] after the clinical interview.

Subsample 1 included 10 female patients, aged 20 to 40 years. All of them were diagnosed with depression and personality disorder (four of them with narcissistic personality disorder, three with dependent personality disorder, two with avoidant personality disorder and one with obsessive-compulsive personality disorder). The therapies were conducted by 6 female and 2 male therapists (aged 33 to 45 years; between 4 and 17 years of practical professional experience). The patient of subsample 2 was male, 38 years old at the beginning of therapy, and was diagnosed with panic disorder, agoraphobia, depression, and dependent personality disorder. He was treated by a female therapist (aged 35 years, 9 years of practical professional experience).

Patients were extensively informed prior to commencing therapy and provided written informed consent concerning the use of their data for research purposes. Procedures for this study were approved by the University of Leipzig Ethics Commission.

### Measuring instrument

We used the Intrex questionnaire [24,25] for continuous assessment of the therapeutic interaction which was developed on the basis of the "Structural Analysis of Social Behavior" model [26]. SASB is one of the most influential approaches for the categorization of interpersonal interactions and has been subject to considerable empirical examination and wide application [27]. SASB is a circumplex model which contends that variables that measure interpersonal relations are arranged around a circle in a two-dimensional space. A circle is defined by a horizontal dimension of affiliation and a vertical dimension of interdependence. In the model there are three circles, one for each of three foci of action: The transitive focus captures behavior with which an actor attempts to influence an interaction partner, the intransitive focus describes the reactive behavior of the actor, and the level of the introject reflects the way in which a person interacts with him/herself.

Patients completed the short form of the questionnaire immediately after each therapy session. In each case, they rated the relationship behavior in two different directions ("How did I behave towards my therapist in today's session?" - example: "I clearly and comfortably express my own thoughts and feelings to her." and "How did my therapist behave towards me in today's session?" - example: "She unthinkingly ignored and neglected me.").

We combined the Intrex scores of items 2 to 4 and 6 to 8 for each of the 2 directions and the transitive and intransitive focus to form the weighted affiliation index (see additional file 1*for more information and calculation formula)*. This was proposed by Pincus et al., who showed that a weighted sum of the clusters has attractive distribution characteristics as well as good validity [28]. A high affiliation index signifies an experience of affectionate interaction between patient and therapist.

## Results

### Discussion of the methods previously used for measuring crises

#### The Criterion developed by Stiles et al

Stiles et al. calculated the following four parameters for each individual course using regression analysis: Intercept at the time of midtreatment (midtreatment intercept), the linear trend (slope), the quadratic trend (curve), and the variability (root-mean-square-error, RMSE, which can be understood as the deviation of the raw values from the fitted curve). In order to extract typical change profiles, they computed a cluster analysis using these four parameters. They defined a rupture as "an unusually low score" and determined that there is a rupture-repair episode if an observed value falls at least two times the RMSE below the value predicted by the fitted curve. This general definition was specified by four criteria: 1.) Low scores in the first or the last session are not considered a rupture. 2.) Patients who showed a negative linear trend curve were excluded from the analysis due to the reason that ruptures in the course of a generally deteriorating alliance are never considered to be repaired. 3.) The value of a rupture session has to be less than the value of the previous session in order not to mark ruptures in generally increasing curves. 4.) The value must fall below a pre-specified limit to be defined as a rupture. This specification was necessary for the following reason: Relatively stable courses show a low intraindividual variability (a low RMSE). Thus, a comparatively small deviation from the fitted curve is sufficient to be defined as a rupture. The restriction avoids identifying ruptures in stable courses with permanently high values.

#### The Criterion developed by Strauss et al

The criterion developed by Strauss et al. includes specifications for the rupture and for the repair. Strauss et al. calculated the standard deviation for all individual courses and averaged these scores. They identified a rupture-repair episode if they could find a decrease and a subsequent increase in the curve, which were both at least equal to the averaged standard deviation. They added that this must not be followed by an unrepaired decrease of the same magnitude. Strauss et al. did not explicitly label this latter specification as an exclusion criterion; nonetheless, it implies that repaired ruptures should be ignored entirely if there is an unrepaired rupture afterwards.

#### Sample application of the criteria

We demonstrate the differences of the results between the criteria of Stiles et al. and Strauss et al. in this paragraph. Table 1 contains the number of ruptures measured with both criteria and the number of courses with at least one rupture-repair episode. The total of identified ruptures is considerably different. Using the criterion of Stiles et al., we found 12 ruptures in 7 courses, compared to 23 ruptures in 7 courses with the criterion of Strauss et al. Accordingly, we observed a discrepancy in the course of the long-term therapy (4 vs. 27 ruptures).

**Table 1:** Comparison of the criteria of Stiles et al. and Strauss et al.

	**Stiles**^**a**^	**Strauss**^**b**^
Subsample with 29-35 therapy sessions (n = 10)		
Total RREs	12	23
Courses with at least one RRE	7	7
Courses with no RRE	1	2
Excluded courses	2	1

Single case with 200 therapy sessions (n = 1)		
Total ruptures/RREs	4	27

RRE = Rupture-repair episode.

^a^Stiles = criterion by Stiles et al. [7], two RMSEs; ^b^Strauss = criterion by Strauss et al. [8], one SD.

Three courses either had to be excluded or did not contain a rupture using either the Stiles et al. or the Strauss et al. criteria. Two of them were the same. (In the Stiles et al. procedure two patients had to be excluded because of an overall negative linear trend. Within one course no rupture-repair episode could be identified. This course was highly fluctuating, resulting in a high RMSE and hence a threshold below which no value fell. In the Strauss et al. procedure one case had to be excluded due to an unrepaired rupture in the last session. The other two cases were relatively stable and did not contain a rupture-repair episode.)

### Proposals for modification of the criteria previously used for measuring crises

#### 1 - Threshold for identifying ruptures

Stiles et al. defined a rupture as "an unusually low score" and determined that there is a rupture-repair episode if an observed value falls at least two times the RMSE below the value predicted by the fitted curve. Strauss et al. used the less strict threshold of one standard deviation.

Since the authors used different thresholds they implicitly predefined the magnitude of the crises to be identified. We propose to use both, the stricter (2 sd) and the less strict threshold (1 sd) in order to ensure the comparability of the results of different studies and to analyze the extent of deteriorations in the therapeutic relationship in connection with the outcome.

#### 2 - Interindividual and intraindividual variability

Strauss et al. considered the interindividual variability only: They calculated the standard deviation for all individual courses and averaged these scores. As a result, intraindividually less significant declines in highly fluctuating courses could be marked as ruptures. Stiles et al. considered the intraindividual variability only. As a result, minor fluctuations occurring in relatively stable courses might be overrated and misclassified as rupture-repair episodes. Stiles et al. avoided this problem by establishing the additional condition that the value must be under a predetermined limit to be defined as a rupture. However, it remained unclear how they determined the cut-off. It seemed somewhat arbitrary and limits the application to the questionnaire used by Stiles et al. (Agnew Relationship Measure [29]). Moreover, this procedure implies that slight downward trends in stable courses featuring permanently low values would also be marked as ruptures.

To solve this problem, we propose to combine the intraindividual with the interindividual variability and to favor the stricter of the two values in each case, i.e. to apply the intraindividual standard deviation in high variability profiles (intraindividual > interindividual variability) and to apply the interindividual standard deviation in low variability profiles (intraindividual < interindividual variability). In doing so, we avoid overrating minor fluctuations occurring in relatively stable courses or declines occurring in highly fluctuating courses which are intraindividually less significant.

#### 3 - Exclusion criteria

Stiles et al. noted that ruptures occurring in the course of a generally deteriorating alliance can never be regarded as fully repaired and that patients showing a negative linear trend curve should be excluded from the analysis for this reason. Strauss et al. ignored all repaired ruptures in cases with an unrepaired rupture at the end of treatment and removed these courses from the analysis completely.

These exclusion criteria which call for removing potentially unsuccessful courses from the analysis should be reconsidered, especially in connection with outcome studies, as this approach seems to contain a circular argument. Also, the theoretical content of the exclusion criteria can be challenged. Rupture-repair sequences are not regarded as a separate mechanism of change when using these exclusion criteria. In theory, a rupture without repair at the end of therapy might be in connection with the termination of therapy. Notwithstanding, conflicts may have been treated in the context of previous episodes. We propose to disregard the exclusion criteria.

#### 4 - Adjusting the repair value to the rupture value

In the Strauss et al. criterion, decreases extending over two standard deviation scores or more are considered to be repaired if there is a subsequent increase of just one standard deviation score. This may result in an inaccurate evaluation of the endpoint of an episode, with the misclassification of an insufficient increase as a full repair.

We recommend adjusting the repair value to the rupture value. Decreases which reach or exceed the value of two standard deviations should only be considered to be repaired if the following increase reached the value of two standard deviations as well.

#### 5 - Supplements

We found the following deficiency in the definition of the criteria: Stiles et al. as well as Strauss et al. provided no information as to how to deal with cases in which two or more consecutive decreases fall below the threshold. We propose to classify these cases as one rupture-repair episode only and to aggregate all session-to-session differences after the first rupture mark. It was equally unclear at what point these cases should be classified as repaired. Do they have to be repaired twice or does the sum of the two consecutive session ruptures needs to be repaired? Or does only the second rupture session need to be taken into account? In the latter case the extent of the rupture would be ignored or the extent of the repair would be overestimated. We recommend adjusting the repair value to the rupture value as, in our view, the crisis within the therapeutic relationship can only be seen as overcome if the previous level is reached again.

*(*We also determined the effect of the modifications on the number of identified ruptures. Sample application of the modified criteria: *See *additional file 2*)*

### Discussion of an alternative criterion for measuring crises

#### The crisis-repair criterion

We developed an alternative criterion which is able to include rupture patterns which had not been considered before as well as the length of the rupture-repair episodes. It is based on summing up the differences of session-to-session values until the point where the direction change is reached. (Constant values are not regarded as a direction change. Sessions with constant values are classified as belonging to the rupture, instead of belonging to the repair. This specification may be of importance when determining the length of a rupture or repair.) With this procedure, it is possible to take into account ruptures that develop in the form of gradual downward trends. Moreover, this procedure allows us to determine the beginning and the end of a decline more exactly.

We determined that a rupture must be identified as taking place if the summation value of a decline reaches at least one respectively two standard deviations. We defined a rupture as repaired if there is a positive difference between the start value and a subsequent value or a difference value that lies within the respective standard deviation. As described in the modification 2 paragraph we compared the intraindividual with the interindividual standard deviation; in each case, we favored the stricter of the two values. We referred to the suggested alternative criterion as the "crisis-repair criterion" to imply that we do not only include leaps (ruptures), but also take gradual downward trends into account.

#### Sample application of the criterion

Comparing the results of the crisis-repair criterion with the criteria of Stiles et al. and Strauss et al., there is a considerable difference in the number of ruptures (Table 2). We detected a total of 35 rupture-repair episodes within 9 of the shorter courses and 34 episodes within the long-term therapy when using a threshold of 1 standard deviation score. Applying the stricter value of 2 standard deviation scores, we identified 15 episodes in 7 shorter courses and 9 in the long-term course. (Within the long term course of 200 therapy sessions, we used the intraindividual value solely (SD 41.46) in order not to merge the values of therapies with different lengths. The interindividual averaged SD value of the subsample with 29-35 therapy sessions was 38.69.)

**Table 2:** Comparison of the crisis-repair criterion with the criteria of Stiles et al. and Strauss et al.

	**Crisis-repair criterion 1 SD**^**a**^	**Crisis-repair criterion 2 SD**^**b**^	**Stiles**^**c**^	**Strauss**^**d**^
Subsample with 29-35 therapy sessions (n = 10)				
Total CREs/RREs	35	15	12	23
Courses with at least one CRE/RRE	9	7	7	7
Courses with no CRE/RRE	1	3	1	2
Excluded courses	-	-	2	1

Single case with 200 therapy sessions (n = 1)				
Total CREs/RREs	34	9	4	27

CRE = Crisis-repair episode; RRE = Rupture-repair episode.

^a^Crisis-repair criterion one SD; ^b^Crisis-repair criterion two SDs; ^c^Stiles = criterion by Stiles et al. [7], two RMSEs; ^d^Strauss = criterion by Strauss et al. [8], one SD.

**Figure 1:**
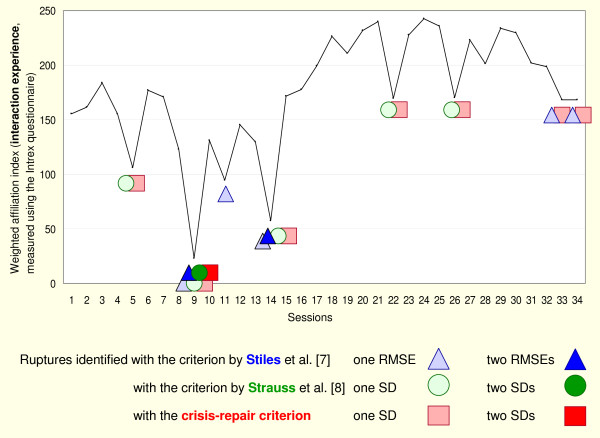
**How the choice of a criterion influences the findings**. Example - patient X.

Figure 1 shows an example of how the choice of a criterion influences the findings.

### Classification of theoretical patterns of crisis-repair sequences

#### Theoretical patterns of crisis-repair sequences

Based on theoretical considerations, we characterized five typical crisis-repair patterns that occur in the progress of therapy. We primarily distinguished the subtypes on the basis of whether deteriorations or repairs of the therapeutic relationship comprise one session or more than one session.

We distinguished the following subtypes:

Pattern 1 ("jump in - jump out", V-shape): Deteriorations developing in leaps from one session to the next comprising one session only that are repaired in the following session.

Pattern 2 ("jump in - slide out"): Deteriorations developing in leaps from one session to the next comprising one session only that are repaired gradually, meaning that the repair comprises more than one session.

Pattern 3 ("slide in - jump out"): Deteriorations developing gradually over more than one session and repaired in the form of leaps from one session to the next.

Pattern 4 ("slide in - slide out"): Deteriorations developing gradually over more than one session and repaired gradually, meaning that the repair comprises more than one session.

Pattern 5 ("complex pattern"): Sequences in which two or more ruptures are separated by a change of direction.

**Figure 2:**
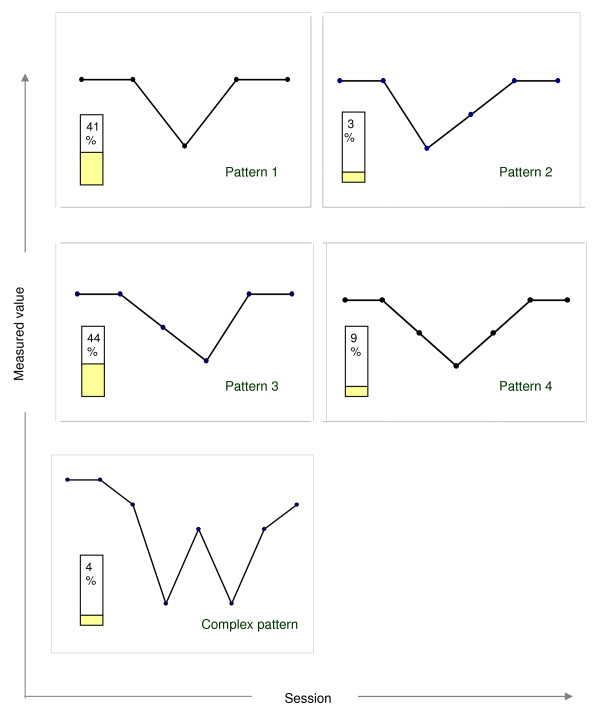
**Classification of theoretical patterns of crisis-repair sequences**. Schematic picture of the five patterns and the frequencies of their occurrence. Pattern 1 ("jump in - jump out", V-form): Deteriorations developing in leaps from one session to the next, comprising one session only and being repaired in the following session. Pattern 2 ("jump in - slide out"): Deteriorations developing in leaps from one session to the next, comprising one session only. Gradual repair, comprising more than one session (at least two). Pattern 3 ("slide in - jump out"): Deteriorations developing gradually over more than one session, being repaired in the following session. Pattern 4 ("slide in - slide out"): Deteriorations developing gradually over more than one session, being repaired gradually (repair comprises more than one session). Pattern 5 ("complex pattern"): Sequences in which two or more ruptures are separated by a change of direction. (Crisis events occurring within single sessions were not taken into consideration).

The classified subtypes of crisis-repair episodes are depicted schematically in Figure 2.

#### Frequency distribution of the theoretical patterns and length of the episodes

We assigned each episode calculated with the crisis-repair criterion to one of the theoretical types and ascertained the frequency distribution of the types. Table 3 and Figure 2 contain the frequency of occurrence of the subtypes of crisis-repair episodes.

**Table 3:** Frequency distribution of the theoretical patterns of crisis-repair episodes

Patterns					
	
	**1**^**a**^	**2**^**b**^	**3 (3a, 3b)**^**c**^	**4 (4a, 4b)**^**d**^	**complex**^**e**^
Subsample with 29-35 therapy sessions (n = 10)					
Total CREs	12	2	17 (4, 13)	3 (1, 2)	1
%	34.3	5.7	48.6 (11.4, 37.1)	8.6 (2.9, 5.7)	2.9
Courses with at least one CRE	6	2	8 (2, 6)	3 (1, 2)	1

Single case with 200 therapy sessions (n = 1)					
Total CREs	16	0	13 (4, 9)	3 (0, 3)	2
%	47.1	0	38.2 (11.8, 26.5)	8.8 (0, 8.8)	5.9

CRE = Crisis-repair episode; % = Percentage among all CREs.

^a^Pattern 1 = jump in - jump out; ^b^Pattern 2 = jump in - slide out; ^c^Pattern 3 = slide in - jump out; (3a) = slide in without a jump (gradual downward trend only); (3b) = slide in including a jump; ^d^Pattern 4 = slide in - slide out; (4a) = slide in without a jump (gradual downward trend only); (4b) = slide in including a jump; ^e^Complex pattern = Two or more ruptures are intermitted through a change of direction.

In those cases in which deterioration occurred over more than one session ("slide in", patterns 3 and 4) we further specified whether the deterioration arose from gradual downward trends only ("slide in without a jump", patterns 3a and 4a) or in combination with one or more sudden sharp declines ("slide in including one or more jumps", patterns 3b and 4b). We did not subdivide cases with a repair extending over more than one sessions (patterns 2 and 4) as these cases occurred very rarely.

The most frequent pattern was the simple V-shape ("jump in - jump out", pattern 1, 28 ruptures, 40.6%). The second most common pattern was a decline over more than one session including a jump with a sudden repair ("slide in including one or more jumps - jump out", pattern 3b, 22 cases, 31.9%). These two patterns together account for nearly three quarters of the crisis-repair episodes in our sample. Patterns with a sliding repair occurred significantly less frequently ("slide out", patterns 2 and 4, 8 cases, 11.6%). The complex pattern was even rarer (3 cases, 4.3%).

Sudden declines and increases in the value of the therapeutic relationship variables (leaps) are a central element of therapeutic progress. 57 of a total of 66 declines (86.4%) included at least one jump. Three quarters of the declines extending over more than one session included at least one jump.

Our criterion enables us to measure the beginning and the length of downward and upward trends exactly. This makes it possible to determine how many sessions the crises and repairs lasted. Within the complete sample, 30 deteriorations of the therapeutic relationship comprised 1 session only (45.4%), 25 cases (37.9%) extended over 2 sessions, 9 declines extended over 3 sessions, 1 extended over 4, and 1 extended over 6 sessions. The repair segments are shorter altogether: Within the complete sample, 58 repairs comprised 1 session only (87.9%), 6 extended over 2 sessions (9.1%), 1 extended over 3 sessions, and 1 extended over 4 sessions. (Complex patterns were not counted for these calculations.)

The results may briefly be summarized as follows: Most of the deteriorations of the therapeutic relationship comprised one or two sessions. Declines extending over more than three sessions occurred infrequently. The longest downward trend comprised a period of six sessions. Almost all repairs occurred within one session. The length of the entire crisis-repair episode for the complete sample averaged 2.9 sessions.

## Discussion

Temporary deteriorations within the therapeutic relationship are a distinctive feature of change over the course of the processes, which was also noted in our sample. The general question we raised is: what crises are useful and which ones will harm the process? To create a systematic access, we proposed to distinguish three characteristics of therapeutic crises: number, magnitude and length.

The number of identified crises varies significantly, depending on the criterion used for measurement. Stiles et al. [13] pointed out that their rupture-repair criterion was developed *ad hoc *and should be regarded as preliminary; they indicated that application to other samples remained necessary. Strauss et al. [14] remarked that their method of quantifying alliance ruptures "is most similar to that of Stiles et al. (2004)" (p. 343). However, our testing yielded differing results, depending on the criterion that was used. Thus, the comparability of the results of studies which used different criteria seems to be limited in terms of the frequency of the ruptures and the ascertained number of courses with at least one rupture.

Stiles et al. and Strauss et al. used different thresholds for identifying ruptures. In doing so, they implicitly predefined at which magnitude a crisis is identified as one. We propose to use both, the stricter (2 sd) and the less strict threshold (1 sd) in order to ensure the comparability of the results of different studies and for the purpose of analyzing the extent of deteriorations in the therapeutic relationship in connection with the outcome.

We noticed weak points in the criteria definition of Stiles et al. and Strauss et al. and suggested several modifications. We propose to combine the intraindividual with the interindividual variability and to favor the stricter of the two values in each case, in order to avoid overrating minor fluctuations occurring in relatively stable courses or declines occurring in highly fluctuating courses which are intraindividually less significant. We propose to disregard the exclusion criteria, especially in connection with outcome studies, as this approach seems to contain a circular argument. We propose to classify cases in which two or more consecutive decreases fall below the threshold as one rupture-repair episode only. We recommend adjusting the repair value to the rupture value as, in our view, the crisis within the therapeutic relationship can only be seen as overcome if the previous level is reached again.

In comparison with the criteria of Stiles et al. and Strauss et al., more ruptures were identified with the crisis-repair criterion proposed by us. Using our criterion, it becomes possible to identify crises developing in small steps over a number of sessions; in this way, we also captured gradual downward trends.

We differentiated five crisis-repair subtypes. The most frequent pattern was the simple V-shape ("jump in - jump out", 40.6%). The second most common pattern was a decline over more than one session including a jump with a sudden repair ("slide in including one or more jumps - jump out", 31.9%). It was striking that the episodes in more than half the cases showed more complex progress than a simple high-low-high pattern. It appeared that both the crises and the repairs may extend over several sessions. The length of the crises in the complete sample averaged 1.8 and the length of the repairs 1.2 sessions. The longest downward trend comprised a period of 6 sessions. In this light, we can assume that crisis-repair episodes exist at different temporal levels in the process. Like other authors proved, ruptures can be found within a single session that are repaired before the end of the same session [8-11]. Besides this, there are ruptures that are repaired in the following session (which results in a high-low-high pattern) and there are ruptures that extend over several sessions. Stiles et al. remarked that their criteria "were crude, justified by making use of ratings that were gathered only once for each session". They further stated that "moment-by-moment ratings of the session process are potentially much more sensitive, albeit more laborious" (p. 91). Alternatively, we can assume that the length of the episodes is related to specific characteristics of the therapeutic process. For example, it can be hypothesized that there is a relation to the extent of the entanglements of the therapists with a pathological hostile interaction pattern of the patient. Prolonged crises may potentially occur in more disturbed patients with severe interpersonal problems. Less severe interpersonal problems may lead to more subtle crises that may be resolved within the same or at least within the next session. It is also conceivable that the length of the episodes is negatively related to therapeutic qualities such as the professional experience.

As a result of the present observations, it would be interesting for future analyses to examine the potential influence of the mentioned variables on the length of the crisis-repair episodes and the effect of the length of the episodes on the outcome. For this purpose it is necessary to define in which session a crisis begins and in which session it should be considered repaired. Moreover, it seems to be useful to exactly define an episode to analyze the sessions in detail in order to reveal the mechanisms behind the crisis-repairs in the course of the therapeutic relationship. To create a basis for this kind of objective, it makes sense to examine the criteria that determine what a rupture or what a rupture-repair sequence is in depth.

At least one jump was seen in 86.4% of the declines. Thus, discontinuous sudden declines and increases in the trajectories of the therapeutic relationship are a central element of therapeutic progress. However, it also must be stated that we found gradual downward trends (isolated or in combination with a jump) in more than half the cases. When looking at the repairs, we can state that the gradual trend played a less important role. We found an isolated jump in 84% of the cases which lets us conclude that a breach in the therapeutic relationship can be dissolved completely immediately after focusing in most cases.

We have shown that the choice of a criterion influences the findings. We can not draw any unambiguous conclusions about which is the best methodology to use but we may provide support to researchers in making an informed and thoughtful decision about how to proceed, depending on the context. Strictly speaking, it must be decided beforehand whether leaps (discontinuities) or crises of varying length can be assumed to be the crucial mechanism of change. If we theoretically view the leap to be crucial, the modified criterion of Strauss et al. could be used. If we theoretically focus on the importance of extreme lows, the modified criterion of Stiles et al. appears to be useful. The advantage of using the criterion proposed by us is that ruptures with different patterns and lengths (including gradual trends) are included. This means we regard all crises, both gradual and sudden deteriorations, in the experienced therapeutic relationship as important mechanisms of change without limiting our scope to single discontinuities, i.e. sudden deteriorations. This provides a wider range for different assumptions.

We would like to point out that it is useful to ascertain variables continuously throughout the processes. The results may be distorted if only selected sessions are examined as jumps between the measurements can occur. The study of Strauss et al. was conducted with larger intervals between the measurements. A part of the ruptures found in the study of Strauss et al. may have been developed gradually over several sessions or the number of ruptures may be even higher than documented.

It also should be mentioned that the number of identified ruptures might depend on the measuring instrument. Stiles et al. used the Agnew Relationship Measure [29], while Strauss et al. used the California Psychotherapy Alliance Scale [30]. It is possible that certain different aspects of the therapeutic relationship are detected by different instruments. It might be that the Intrex [24,25] is a particularly sensitive instrument for showing fluctuations in the therapeutic relationship as it measures patterns of the therapeutic relationship (the interaction between therapist and patient) directly and in a relatively differentiated manner.

In our present state of knowledge, many questions still remain open. Further analyses on this topic are required. This may be unsatisfactory for the time being, as we can not provide hard data at this first stage. Nevertheless, our approach can contribute to pave the way for further steps in this kind of research thoroughly and with a solid grounding. A phenomenological discussion of the conception of crises is useful in order to distinguish specific aspects and to specifically link these characteristics to the treatment outcome in future studies. This kind of systematic research offers the advantage that conclusions about both, positive and negative correlations can be drawn in the future. Empirical studies of the therapeutic relationship and of the factors influencing that relationship are essential to clinical practice as change always emerges from the context of the patient-therapist relationship independently of the applied therapeutic technique. The findings of the study give an insight into basic mechanisms of change within the therapeutic relationship. After all, the methodological concerns we address might be adaptable to different research areas where the analysis of fluctuations in a variable of interest over time is relevant.

## Conclusions

To create a systematic access we proposed to basically distinguish therapeutic crises in the process with regard to three features: number, magnitude and length. These characteristics can be related to the therapeutic outcome individually, in order to address the question, which crises are useful and which crises will harm the process.

The number of identified rupture-repair episodes varies significantly depending on the measuring criterion. The methods previously used (Stiles et al. and Strauss et al.) applied different thresholds for identifying ruptures. Thus, they implicitly predefined the magnitude of the crises to be identified. So far, the effect of the length of the crises has been disregarded in previous studies. We noticed some weaknesses in the criteria definition of Stiles et al. and Strauss et al. and recommended several modifications.

In comparison with the criteria of Stiles et al. and Strauss et al., more ruptures were identified with the crisis-repair criterion proposed by us. Using our criterion, it becomes possible to identify crises developing in small steps over a number of sessions. That means we regard all crises, both gradual and sudden deteriorations in the therapeutic relationship, as important mechanisms of change as opposed to focusing on single discontinuities, i.e. sudden deteriorations. This leaves more scope for assumptions.

We classified different theoretical patterns of rupture-repair sequences in therapy processes. The most frequent pattern was the simple V-shape (40.6%). The second most common pattern was a decline over more than one session including a jump with a sudden repair (31.9%). The longest downward trend comprised a period of six sessions. We conclude that crises extend over different temporal levels in the process. When looking at the repairs, we found an isolated jump in 84% of the cases. We conclude that a breach in the therapeutic relationship can be dissolved completely immediately after focusing, in most cases.

## Competing interests

The authors declare that they have no competing interests.

## Authors' contributions

AG conceived of the study, participated in its coordination and drafted the manuscript. EB, MG and RE contributed to the discussions about the topic. RE drafted the manuscript. All authors read and approved the final manuscript.

## Pre-publication history

The pre-publication history for this paper can be accessed here:

http://www.biomedcentral.com/1471-2288/12/10/prepub

## Supplementary Material

Additional file 1 **Calculation of the affiliation index**. The file contains more information on the weighted affiliation index and its calculation formula. Click here for file

Additional file 2 **Comparison of the Stiles et al. and Strauss et al. criteria with their modifications**. Sample application of the modified criteria - the file contains two tables showing the effect of the modifications of the Stiles et al. and Strauss et al. criteria on the number of identified ruptures. Click here for file
