# Supplementary material for: Crisis-repair sequences - considerations on the classification and assessment of breaches in the therapeutic relationship
Source: BMC Med Res Methodol. 2012 Feb 3;12:10. doi: 10.1186/1471-2288-12-10 (PMC3320522; doi:10.1186/1471-2288-12-10)
Supplement: Additional file 1 — Calculation of the affiliation index. The file contains more information on the weighted affiliation index and its calculation formula. [file 1471-2288-12-10-S1.DOC]

**Additional file 1 - Calculation of the affiliation index**

$$A=\frac{4,5\times item2+7,8\times item3+4,5\times item4-4,5\times item6-7,8\times item7-4,5\times item8}{\text{number of incorporated items}}$$

The two items which are directly on the affiliation axis within the circumplex model (Items 3 and 7) contribute more strongly to the weighted affiliation index. Weighted affiliation values range from -280 to 280. We did not distinguish according to focus or direction in our analysis. The value is thus based on 24 items.

(We disregarded the control index since this index is, in our view, more strongly dependent on individual psychopathology and less clearly interpretable in terms of therapeutic progress. [27] have suggested that hostility can be seen as a central component of psychopathology. In almost all investigations, negative values on the affiliation axis have been found to be significantly related to symptoms and diagnoses. According to the authors, the significance of the control axis for the definition of psychopathology is, in contrast, less well-established.)
