# Supplementary material for: Crisis-repair sequences - considerations on the classification and assessment of breaches in the therapeutic relationship
Source: BMC Med Res Methodol. 2012 Feb 3;12:10. doi: 10.1186/1471-2288-12-10 (PMC3320522; doi:10.1186/1471-2288-12-10)
Supplement: Additional file 2 — Comparison of the Stiles et al. and Strauss et al. criteria with their modifications. Sample application of the modified criteria - the file contains two tables showing the effect of the modifications of the Stiles et al. and Strauss et al. criteria on the number of identified ruptures. [file 1471-2288-12-10-S2.DOC]

**Additional file 2 - Comparison of the Stiles et al. and Strauss et al. criteria with their modifications**

Comparison of the Stiles et al. criterion with its modifications

|  | Stiles | Modifications | | |
| --- | --- | --- | --- | --- |
|  |  | 1^a^ | 2^b^ | 3^c^ |
| Subsample with 29-35 therapy sessions (n = 10) | | | | |
| Total ruptures | 12 | 26 | 9 | 14 |
| Courses with at least one rupture | 7 | 8 | 6 | 9 |
| Courses without rupture | 1 | 0 | 2 | 1 |
| Excluded courses | 2 | 2 | 2 | - |
| Single case with 200 therapy sessions (n = 1) | | | | |
| Total ruptures | 4 | 26 | - | - |

^a^Modification to one RMSE threshold for identifying ruptures. ^b^Including the interindividual in addition to the interindividual variability favoring the individually stricter value (applied to the subsample with 29-35 therapy sessions only). ^c^Inclusion of cases excluded with the original criterion because of a negative linear trend.

Comparison of the Strauss et al. criterion with its modifications

|  | Strauss | Modifications | | | |
| --- | --- | --- | --- | --- | --- |
|  |  | 1^a^ | 2^b^ | 3^c^ | 4^d^ |
| Subsample with 29-35 therapy sessions (n = 10) | | | | | |
| Total RREs | 23 | 9 | 23 | 31 | 20 |
| Courses with at least one RRE | 7 | 6 | 7 | 8 | 6 |
| Courses without RRE | 2 | 2 | 2 | 2 | 2 |
| Excluded courses | 1 | 2 | 1 | - | 2 |
| Single case with 200 therapy sessions (n = 1) | | | | | |
| Total RREs | 27 | 6 | - | - | 21 |

*Note*. RRE = Rupture-repair episode.

^a^Modification to one-SD threshold for identifying ruptures. ^b^Including the intraindividual SD in addition to the interindividual SD favoring the individually stricter value (applied to the subsample with 29-35 therapy sessions only). ^c^Inclusion of cases excluded with the original criterion because of an unrepaired rupture at the end of treatment. ^d^Adjusting the repair value to the rupture value.
